# Editorial: Bench to bedside: AI and remote patient monitoring

**DOI:** 10.3389/fdgth.2025.1584443

**Published:** 2025-03-11

**Authors:** Ramin Ramezani, Saam Iranmanesh, Arash Naeim, Peyman Benharash

**Affiliations:** ^1^ Computer Science Department, University of California, Los Angeles, Los Angeles, CA, United States; ^2^ Department of Electrical and Electronic Engineering, Imperial College London, London, United Kingdom; ^3^ David Geffen School of Medicine, University of California, Los Angeles, Los Angeles, CA, United States

**Keywords:** artificial intelligence, remote patient monitoring (RPM), cloud computing, personalized & precision medicine (PPM), machine learning methods

**Editorial on the Research Topic**
Bench to bedside: AI and remote patient monitoring

## Introduction

A comprehensive understanding of patients’ functional and mental well-being is crucial, particularly for individuals with high-risk conditions. Continuous, long-term data collection facilitates improved risk stratification, personalized treatment selection, and early detection of adverse events. Real-time monitoring enables timely interventions, reducing resource utilization while enhancing quality of life. As healthcare systems transition toward precision and accountable care, achieving these goals necessitates innovative, cost-efficient, and scalable patient monitoring technologies.

Recent advancements in mobile applications, wearable devices, and physiological sensors have enabled continuous digital monitoring of a wide range of physiological and psychological parameters, often referred to as “digital breadcrumbs.” This editorial collection explores how these technologies enhance clinical decision-making, the challenges in data analysis, and methods to bridge the gap from research to real-world implementation with the goal that contributions to this call will further facilitate the “translation” of clinical use of technology from “bench to bedside”.

### Summary of the published articles

The COVID-19 pandemic significantly accelerated the adoption of telemedicine and remote patient monitoring. The study (Pugmire et al.) examined a healthcare provider's experience in launching a COVID-19 Remote Patient Monitoring (CRPM) program and found that leadership engagement was the most frequently cited key factor for successful implementation.

The pandemic is believed to have at least partly contributed to the global rise in mental health disorders, with the CDC estimating that one in three adults in the U.S. experiences significant anxiety or depression symptoms. While ketamine has emerged as a major innovation in psychopharmacology in recent years, the FDA approved a specific form of ketamine, Spravato, for use in Treatment-Resistant Depression (TRD) and Major Depressive Disorder (MDD) with suicidal ideation. While these drugs show promise, a substantial degree of patient monitoring is critical for treatments that alter consciousness. The study (Solomon et al.) explored a proof-of-concept system, the MindMed Session Monitoring System, which passively collects physiological data such as activity levels, steps, audio, and heart rate from mobile and wearable devices. The study assessed data quality and feasibility in 24 participants undergoing Spravato (ketamine) treatment, providing insights into the potential for real-time monitoring applications in mental health care.

With the rapid increase in the geriatric population (65+) globally, including the U.S., alongside the staggering number of approximately 5 million patients in the U.S. living with dementia, the importance of remote patient monitoring has become increasingly evident. Monitoring Activities of Daily Living (ADLs) through such technologies represents one of their most suitable applications; however, certain limitations persist. These include challenges in capturing detailed intra- and inter-individual variabilities, as well as concerns regarding subjectivity, reliability, reproducibility, and sensitivity to changes in behavior.

The paper (Narayan et al.) presents pilot data on an AI-based clinical decision support tool that leverages computer vision to monitor older adults in their home environments. The authors express optimism that future advancements in monitoring technologies will enable the accurate assessment of ADLs with minimal representative samples, thereby improving behavioral analysis models and enhancing their ability to determine functional performance in various environments with greater accuracy and objectivity.

Paper (Armstrong et al.) introduces a novel method for automated biomarker identification and quantification using standard RGB video capture for monitoring low-risk patients, such as those with osteoarthritis (OA). By leveraging a camera-based approach, this method facilitates the monitoring of treatment effectiveness and disease progression, thereby reducing the burden on healthcare providers, particularly when routine in-person visits for low-risk patients may be challenging to sustain. The study collected clinically relevant motion data from 20 patients and proposed a technique that generates 3D human shape and pose from 2D video data through adversarial training in a deep neural network with a self-attention mechanism designed to encode both spatial and temporal information. In a clinical study on a small population of patients with knee pain, the extracted biomarkers demonstrated statistical significance in evaluating treatment outcomes, tracking rehabilitation progress, and monitoring disease progression. Key biomarkers identified include the cumulative acceleration of elbow flexion/extension during a sit-to-stand task, as well as the smoothness of knee and elbow flexion/extension during both squat and sit-to-stand movements.

Paper (Rayan et al.) examines the experiences, barriers, and expectations associated with deploying remote patient monitoring (RPM) systems for intensive care unit (ICU) nurses in a university hospital setting. The study conducted interviews with ICU nurses to assess their perspectives on the use of technologies such as vital signs monitors, electrocardiogram (ECG) machines, and pulse oximeters, which facilitate continuous monitoring of patients' physiological indicators.

Nurses emphasized the importance of user-friendly interfaces and clear visualization in enhancing the efficiency of these systems. However, a key concern identified was poor alarm handling, which was recognized as a potential patient safety hazard. The study underscores the critical need for high accessibility, wireless, noninvasive, and interoperable RPM systems, which would likely benefit from the expansion and deployment of cloud-based platforms to improve integration and usability in clinical workflows.

Among the wide range of remote patient monitoring (RPM) applications, various gadgets and devices have been developed for cough detection, a symptom commonly associated with respiratory diseases such as asthma and chronic obstructive pulmonary disorder (COPD). Research in this area has primarily focused on machine learning and deep learning algorithms trained on audio signals. However, the study described in (Diab and Rodriguez-Villegas) proposes an alternative approach using a non-contact tri-axial accelerometer to differentiate between cough and non-cough events/movements. A key advantage of this method is the widespread presence of accelerometers in modern mobile and wearable devices, making it a potentially scalable alternative. Additionally, it addresses privacy and security concerns often raised by audio-based recording devices. The study utilized feature extraction techniques combined with logistic regression classification, achieving approximately 90% accuracy, sensitivity, and F1 score. These results demonstrate the feasibility of a motion-based wearable cough detector, suggesting its potential for future implementation.

Dealing with datasets from real-world healthcare scenarios often involves addressing imbalanced datasets, a common challenge in machine learning and classification tasks, where medically significant yet rare events occur infrequently. Apnoea serves as an example of such rare medical events. The study (Abdulsadig and Rodriguez-Villegas) explores class rebalancing techniques as a means to mitigate dataset imbalance. The study investigates 10 commonly used imbalance mitigation methods, including the Synthetic Minority Oversampling Technique (SMOTE), to improve the detection of apnoea events from photoplethysmography (PPG) signals acquired from the neck. The findings indicate that while random undersampling (RandUS) enhances sensitivity scores, it harms the overall accuracy due to the reduction in training sample size. The study underscores the need for further research into artificial data generation techniques, particularly considering subject dependencies, which have been a notable challenge in the apnoea dataset.

Mortality from non-communicable diseases (NCDs) is increasing at an alarming rate worldwide. These diseases account for 35 million deaths annually, with 14% attributed to cardiovascular diseases. As a result, the need for monitoring patients outside clinical settings is becoming increasingly evident. The study (Kassaw et al.) investigates the willingness of 397 participants to adopt remote patient monitoring (RPM) through an interview-based questionnaire. The findings indicate that age, mobile phone ownership, and perceived usefulness are significantly associated with a participant's inclination to use RPM technologies.

While much of the research in this journal, as well as broader developments in remote patient monitoring (RPM), focuses on building sensors and systems to collect biomarkers, physiological, and functional parameters, these studies also emphasize the need for accessible and robust infrastructures. Such infrastructures must efficiently support scalability, data security, and reliable data processing to fully harness the potential of RPM technologies outside traditional healthcare settings. Cloud-based infrastructures offer scalability, security, and real-time data processing, making them ideal for large-scale RPM deployment. Unlike research studies with limited patient populations, cloud-based platforms can support millions of users, enabling real-time alerts for timely clinical interventions while reducing costs. Paper (Cao et al.) presents an IoT-based healthcare architecture utilizing Microsoft Azure and AWS to build HIPAA-compliant RPM systems. By leveraging cloud scalability, security, and load balancing, the study demonstrates how these platforms facilitate efficient health monitoring. Additionally, it provides a cost analysis and performance evaluation of Azure and AWS, highlighting their feasibility for large-scale healthcare applications.

With the increasing adoption of cloud-based infrastructures and the Internet of Things (IoT) in medical and remote patient monitoring, the term Internet of Medical Things (IoMT) has emerged, encompassing wearables, gadgets, and sensors used in healthcare. However, security and privacy concerns remain a major challenge due to sophisticated cyber threats and the sensitivity of patient data. Traditional machine learning methods often struggle to capture complex patterns in IoMT data, while conventional intrusion detection systems fail to detect unknown attacks, resulting in high false positive rates and compromised data security. Paper (Shaikh et al.) addresses this issue by proposing an anomaly-based intrusion detection system integrating Random Forest for feature extraction, Convolutional Neural Networks (CNNs) and Long Short-Term Memory (LSTM) models for pattern recognition, and a Self-Adaptive Attention Layer Mechanism (SAALM) tailored for IoMT-specific challenges. The study demonstrates improved accuracy over traditional intrusion detection methods, highlighting its potential to enhance the security and confidentiality of patient data in IoMT healthcare systems.

### Tractions and future directions

As medicine shifts toward personalized care and home-based healthcare, the growing affordability and enhanced capabilities of wearable devices—approaching medical-grade quality—along with smart gadgets, are driving the development of smart homes for remote patient monitoring (RPM). Concurrently, artificial intelligence (AI) is revolutionizing healthcare by enabling disease prediction, patient classification, risk stratification, hospitalization forecasting, and real-time detection of adverse events.

This publication, aimed at translating clinical technology from Bench to Bedside, brings together cutting-edge research on RPM devices, IoT infrastructures, healthcare data analytics, and security. The included studies highlight the growing role of AI in RPM for various medical applications. However, the complexity of the healthcare ecosystem, involving multiple stakeholders and financial constraints, means that technology adoption remains gradual. Despite greater acceptance of digital health solutions post-2019, challenges such as digital literacy and the need for user-friendly interfaces continue to affect patient compliance.

Moreover, while machine learning and predictive algorithms have demonstrated promising results, they still require further refinement to handle imbalanced healthcare datasets and rare medical conditions. Generative AI is expected to play a role in addressing data imbalance challenges. Another significant hurdle in AI applications is the difficulty in identifying general patterns and hypotheses from clinical datasets, given the unique treatment pathways of individual patients. Designing AI-driven systems that offer meaningful clinical recommendations remains a challenge.

Additionally, biases in healthcare datasets, whether inherent or resulting from medical practices, must be carefully examined, as even well-intentioned clinical decisions can introduce unintended bias. Moving forward, rigorous investigation and validation are essential. While the opportunities in this field are immense, researchers must be prepared for significant challenges as they work towards advancing AI-driven healthcare solutions.

In summary, the intersection of AI, wearable devices, and remote patient monitoring is transforming healthcare delivery. As medical technology evolves, several key challenges and opportunities emerge:

• **Scalability and Infrastructure**: Cloud-based solutions will play a crucial role in supporting large-scale remote patient monitoring deployment.
• **Digital Literacy and Compliance**: User-friendly interfaces and patient education to enhance adoption rates and engagement.
• **AI for Personalized Healthcare**: Machine learning models must evolve to handle imbalanced datasets and subject-specific variability.
• **Bias and Ethical Considerations**: Bias in healthcare datasets remains a concern, requiring rigorous validation and fair AI frameworks.
• **Generative AI for Data Augmentation**: The use of generative models may help address data scarcity and improve AI performance in rare medical conditions.

While the future of AI-driven remote monitoring is promising, challenges such as regulatory hurdles, clinical validation, and healthcare system integration must be addressed to fully realize the potential of these technologies.

